# Syncytial nuclear aggregates in normal placenta show increased nuclear condensation, but apoptosis and cytoskeletal redistribution are uncommon

**DOI:** 10.1016/j.placenta.2013.02.007

**Published:** 2013-05

**Authors:** S.J. Coleman, L. Gerza, C.J.P. Jones, C.P. Sibley, J.D. Aplin, A.E.P. Heazell

**Affiliations:** a Institute of Human Development, University of Manchester, Maternal and Fetal Health Research Centre, UK; b St. Mary's Hospital, Central Manchester University Hospitals NHS Foundation Trust, Manchester Academic Health Science Centre, Maternal and Fetal Health Research Centre, Manchester M13 9WL, UK

**Keywords:** Syncytial knots, Syncytial bridges, Apoptosis, Cytoskeletal proteins, Syncytiotrophoblast

## Abstract

**Introduction:**

Syncytial nuclear aggregates (SNAs) are increased in pregnancy complications; however, little is known about their origin or function. This study aimed to characterise SNAs in more detail than has been reported previously.

**Methods:**

Immunohistochemistry and morphological examination at the light and ultrastructural level were used to determine the nature and structure of SNAs.

**Results:**

SNAs comprising bridges and syncytial knots had similar frequency with 974 per mm^3^ of villous tissue (IQR 717–1193) and 833 per mm^3^ (IQR 766–1190), respectively while there were approximately four times as many sectioning artefacts than knots and bridges combined. SNAs had increased proportions of condensed nuclei compared to the remaining syncytiotrophoblast (33.3% vs. 8.9%) and decreased proportions of euchromatic nuclei (0.0% vs. 16.2%), as assessed by examination of an electron micrograph archive. SNAs showed little evidence of apoptosis, with weak positivity for the apoptosis markers M30-neoepitope at 16.6% and TUNEL at 10.0%; strong staining was rarely seen for either marker. Immunofluorescence demonstrated rare association of actin (α, β or γ) with SNAs, whereas tubulin was in close proximity to SNAs and cytokeratin was seen within and surrounding SNAs.

**Discussion:**

M30-positive SNAs traced through serial sections were significantly more likely to be syncytial knots or sectioning artefacts than bridges. Nuclei within SNAs showed signs consistent with degeneration; however, this is unlikely to be an apoptotic process. There are few changes in configuration of cytoskeletal proteins around SNAs.

**Conclusions:**

These data suggest that the biogenesis and functional significance of SNAs still require resolution.

## 1 Introduction

The outer layer of the placenta, the syncytiotrophoblast, is formed by fusion of progenitor cytotrophoblasts into a continuous cell layer [1,2]. The morphology of syncytiotrophoblast nuclei is variable and some are so condensed they are reminiscent of apoptotic nuclei [3,4]. As a result, the hypothesis has arisen that nuclear condensation reflects an ageing process [5–7]. Throughout the third trimester in syncytiotrophoblast, nuclei are found in clusters termed syncytial nuclear aggregates (SNAs) [8]. There are more SNAs in pregnancy complications including preeclampsia, reduced fetal movements, intra-uterine growth restriction (IUGR) and stillbirth than in normal pregnancies [9–13]. The mechanism of nuclear association and the role of SNAs in disease are unknown [14], but electron micrographs suggest cytoskeletal components may be involved in SNA formation or stabilisation [5,15]. Supporting this, the UniGene database lists many actin, tubulin and cytokeratin expressed sequence tags (EST) in placenta (data in Supplementary file 1).

True SNAs have been classified into three groups: sprouts, knots and bridges [4,10,16]. Sprouts, pedunculated collections of euchromatic nuclei generally found in the first trimester placenta, are thought to arise at the initiation of new villi [17,18]. Knots, found towards term, protrude slightly from the villous surface [5]. Bridges are highly nucleated regions that connect two villi [1]. As well as these “true” SNAs, tangential sectioning of the syncytiotrophoblast produces sectioning artefacts [19] that resemble SNAs but are oblique sections through normal syncytium. Aggregates that cannot be reliably classified as knots or bridges, most likely due to misrepresentation of three-dimensional structures in serial two-dimensional sections, are referred to in the present work as “other/unclassified” SNAs.

There is a hypothesis of syncytiotrophoblast turnover that suggests effete nuclei are collected into knots, undergo apoptosis and are shed into the maternal circulation [20–23]. This model is based on i) the condensed morphology of nuclei within SNAs [24], ii) the activation of caspase-8 during cytotrophoblast fusion [25] and iii) the appearance of multinucleate syncytial particles in uterine venous blood [26] and in the lungs of women with terminal eclampsia [27]. However, this hypothesis has been disputed as there is transcriptional activity in the syncytiotrophoblast [28] implying that it is functionally intact and therefore unlikely to be apoptotic. Other research has questioned the role of caspase-8 in cytotrophoblast fusion [29,30], the turnover of effete nuclei into SNAs [4] and whether apoptosis in the syncytiotrophoblast could be controlled apart from within isolated structures [31,32].

This study aimed to investigate by morphometry the nature of SNAs. In line with the above model, it was hypothesised that i) SNAs exist in differing phenotypes, ii) nuclei in SNAs are more condensed than other syncytiotrophoblast nuclei, iii) SNAs show different patterns of cytoskeletal organisation, iv) SNAs are more likely to be positive for markers of caspase-mediated apoptosis than other parts of the syncytiotrophoblast and v) syncytial knots are more likely to be apoptotic than other types of SNA [20–23].

## 2 Methods

Unless otherwise stated, all reagents were obtained from Sigma–Aldrich Chemical Company (Poole, UK). Following ethical approval (North-West Research Ethics Committee 08/H1010/55), fresh placental tissue was obtained following written informed consent. Normal placental tissue was included if it was from 37 to 41 weeks gestation, the baby was in the 10th–90th individualised birthweight centile, maternal BMI was 19–29.9 and there were no maternal morbidities before or during pregnancy. Tissue was collected within 30 min of delivery, wax embedded and sectioned at 5 μm thickness onto 3-aminopropyltriethoxysilane (APES) coated slides.

### 2.1 Tracing SNAs through serial sections

Six placenta were sampled in three areas and ten serial sections were made from these. Sections were stained with haematoxylin and eosin and three fields of view were imaged from each serial section slide. An Open Source programme was developed, Basic Aid Evaluating Serial Sections (BAESS, Version 1.0 [33], available as Supplementary file 2), to track SNAs through serial sections and to total the numbers of manually tagged knots, bridges, other/unclassified SNAs and sectioning artefacts from each set of serial sections. Volume of placental tissue in each serial section stack was estimated by averaging the area measurements of the first and last image and multiplying the area value by 50 μm (10 serial sections of 5 μm).

SNAs were categorised based on their attachment to surrounding villi, developed from Cantle et al. [16]. First, sectioning artefacts were scored as regions appearing to be SNAs on single sections but revealed by serial sections to result from transverse sectioning of syncytiotrophoblast. Second, syncytial knots were scored as regions with ten or more tightly packed nuclei that protruded from a single villus. Third, syncytial bridges were scored as regions composed of 10 or more nuclei linking two villi which, using serial sections, could be observed to separate above and below the bridge. Finally, we scored as other/unclassified any apparent SNA that did not fit into the above categories. Light microscopy staining was visualised using a Dialux 22 microscope (Leitz, Germany) and ImageProPlus 6.0 imaging software (Media Cybernetics Inc.).

### 2.2 Review of electron micrographs

**Fig. 1:**
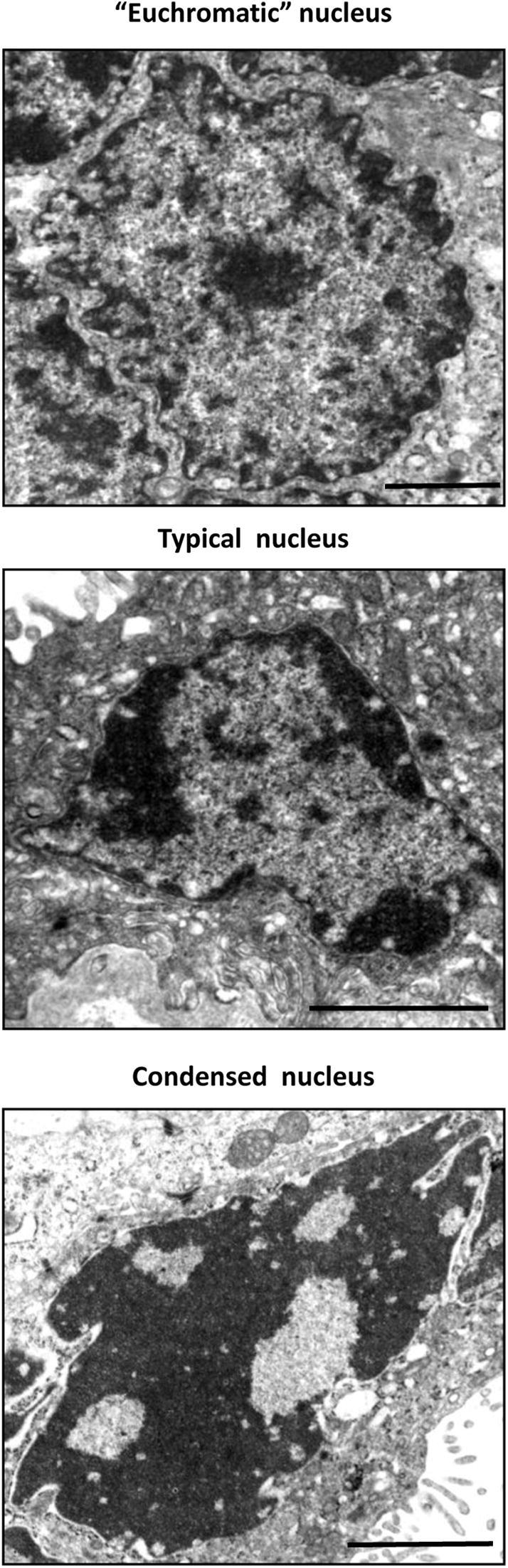
Nuclei were classified into an ordinal scale of patterning. Euchromatic nuclei were defined as nuclei showing very little heterochromatin with the exception of a nucleolus, typical nuclei contain approximately equal amounts of heterochromatin and euchromatin and condensed nuclei contained very little euchromatic matter. Scale bars: 2 μm.

An archive of placental electron micrographs [34] was reviewed to evaluate nuclear morphology in 7 normal placentas. Nuclei in the micrographs were described as being in an SNA if there were ≥10 nuclei in close proximity to one another with little internuclear cytoplasm. As an internal control, SNA nuclei were compared against scores from general syncytiotrophoblast nuclei. Every syncytiotrophoblast nucleus in the micrograph collection was assessed for chromatin patterning according to an ordinal scale as being euchromatic, typical of the syncytiotrophoblast, or condensed (Fig. 1). While truly euchromatic nuclei do not exist in syncytiotrophoblast, the definition was applied to nuclei that appeared mostly euchromatic but with some heterochromatin under the nuclear membrane and dispersed through the nucleoplasm. Data were collected by two blinded investigators (SC, AH) and converted to percentages of the total number of nuclei. Inter-observer variation was calculated by coefficient of variation to be 26.9%.

### 2.3 Immunofluorescence staining for cytoskeletal components

Sections from 3 random areas of 6 normal placentas were blocked for autofluorescence with 1 mg/ml sodium borohydride solution [35] and non-specific binding with 10% animal/2% human serum. Mouse monoclonal anti-α actin (Sigma clone AC-40, 5 μg/ml), anti-β actin (Sigma clone AC-74, 1.25 μg/ml), anti-γ actin (Sigma clone 2-2.1.14.17, 4 μg/ml), anti-α tubulin (Abcam DM1A, 1 μg/ml), anti-β tubulin (Sigma SAP.4G5, 0.46 μg/ml) and anti-cytokeratin 7 (Dako clone OV/TL 12/30, 4.6 μg/ml) were applied overnight at 4 °C. Negative controls were carried out with matching concentrations of isotype matched non-immune mouse IgG. Sections were incubated in secondary antibody rabbit anti-mouse FITC (Dako, 1:200) at room temperature for 1 h then mounted with Vectashield containing DAPI or PI (Vector, Burlingame, CA) to counterstain nuclei. Fluorescence staining was visualised on a Zeiss AxioObserver inverted microscope (Carl Zeiss Inc, Europe) using AxioVision Rel. 4.8.

### 2.4 Assessment of apoptosis

Apoptosis was assessed by terminal d-UTP nick-end labelling (TUNEL) and immunoperoxidase staining for cytokeratin M30-neoepitope as previously described [36,37]. Tissue was blocked with a 3% (*v*/*v*) hydrogen peroxide solution and 10% (*v*/*v*) animal serum against endogenous peroxidise activity and non-specific interactions, respectively. Immunostaining was performed with mouse monoclonal antibody M30 Cytodeath (Roche 1.1 μg/ml) or non-specific mouse IgG1 (1.1 μg/ml) as a negative control and incubated overnight at 4 °C. After washing, biotinylated goat anti-mouse (Dako; 1:200) was applied followed by incubation with avidin–peroxidase (5 μg/ml in 0.125 M Tris buffered saline with 0.347 M NaCl). Immunostaining was revealed by exposure to concentrated 3,3-diaminobenzidine for 3 min. TUNEL staining was performed using a commercially available kit (Roche Applied Diagnostics, Sussex, UK) with modifications to the manufacturers' instructions as previously described [38]. A positive control was treated with DNAse I for 20 min at 37 °C, while a negative control omitted the terminal deoxynucleotidase enzyme. Sections were counterstained with haematoxylin. 10 fields of view were assessed by 2 investigators (LG, AH) per experiment for presence of staining within SNAs, with 0 = no staining, 1 = weak staining around one nucleus and 2 = strong staining around one or more nuclei. Serial sections from normal placentas were similarly stained for M30-neoepitope. M30-positive SNAs were traced through 10 serial sections to determine to which category of SNA they belonged. Immunoreactivity was undetectable in all negative control samples.

### 2.5 Statistical analysis

Statistical significance was assessed using Graphpad Prism (Version 5.03, La Jolla, CA). Kruskal–Wallis test with Dunn's post-hoc test was used as normal distribution cannot be assumed. *P* values of ≤0.05 were assumed to be statistically significant.

## 3 Results

**Fig. 2:**
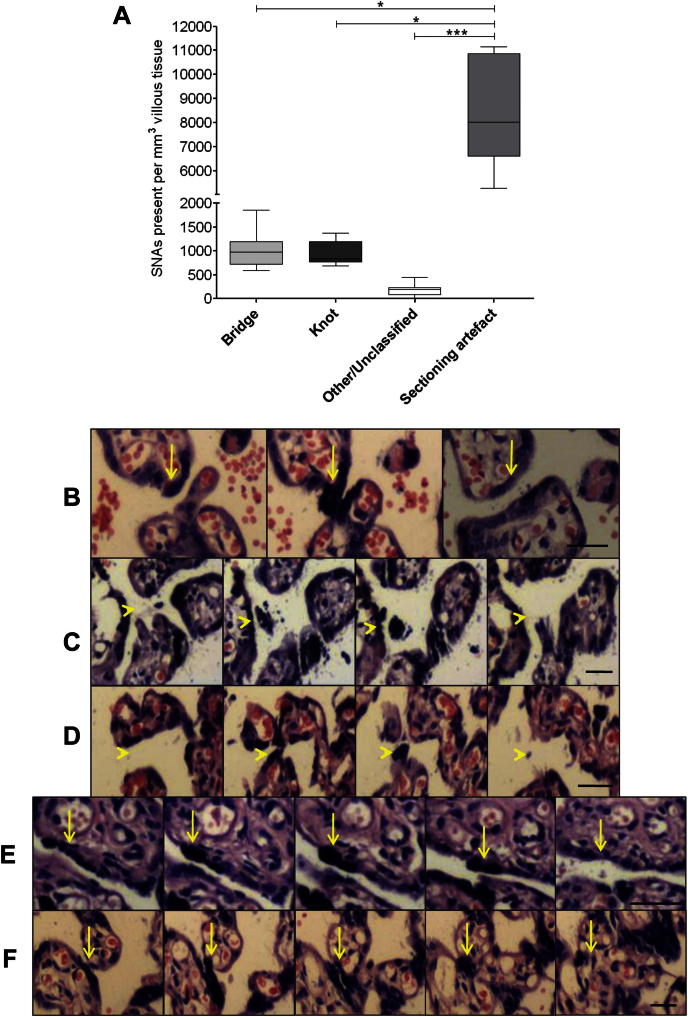
SNAs on single sections were traced through serial sections to determine to which category they belonged. While syncytial knots, bridges and other/unclassified SNAs were present, there were significantly more sectioning artefacts than “true” SNAs (A). Examples are given of a syncytial bridge (B), other/unclassified SNAs (C and D), a knot (E) and a sectioning artefact (F) shown through serial sections, yellow arrows: SNAs. *P ≤ 0.05, ***P ≤ 0.001 as assessed by Kruskal–Wallis followed by Dunn's multiple comparison test. Scale bars: 25 μm.

Sectioning artefacts were the most common phenomenon observed in single sections (Fig. 2A) with over 4-fold greater frequency than other SNA types combined. Syncytial bridges and syncytial knots were more prevalent than SNAs which could not be classified. Examples of SNAs in serial sections are shown, a syncytial bridge (Fig. 2B), other/unclassified (Fig. 2C and D), syncytial knot (Fig. 2E) and sectioning artefact (Fig. 2F).

**Fig. 3:**
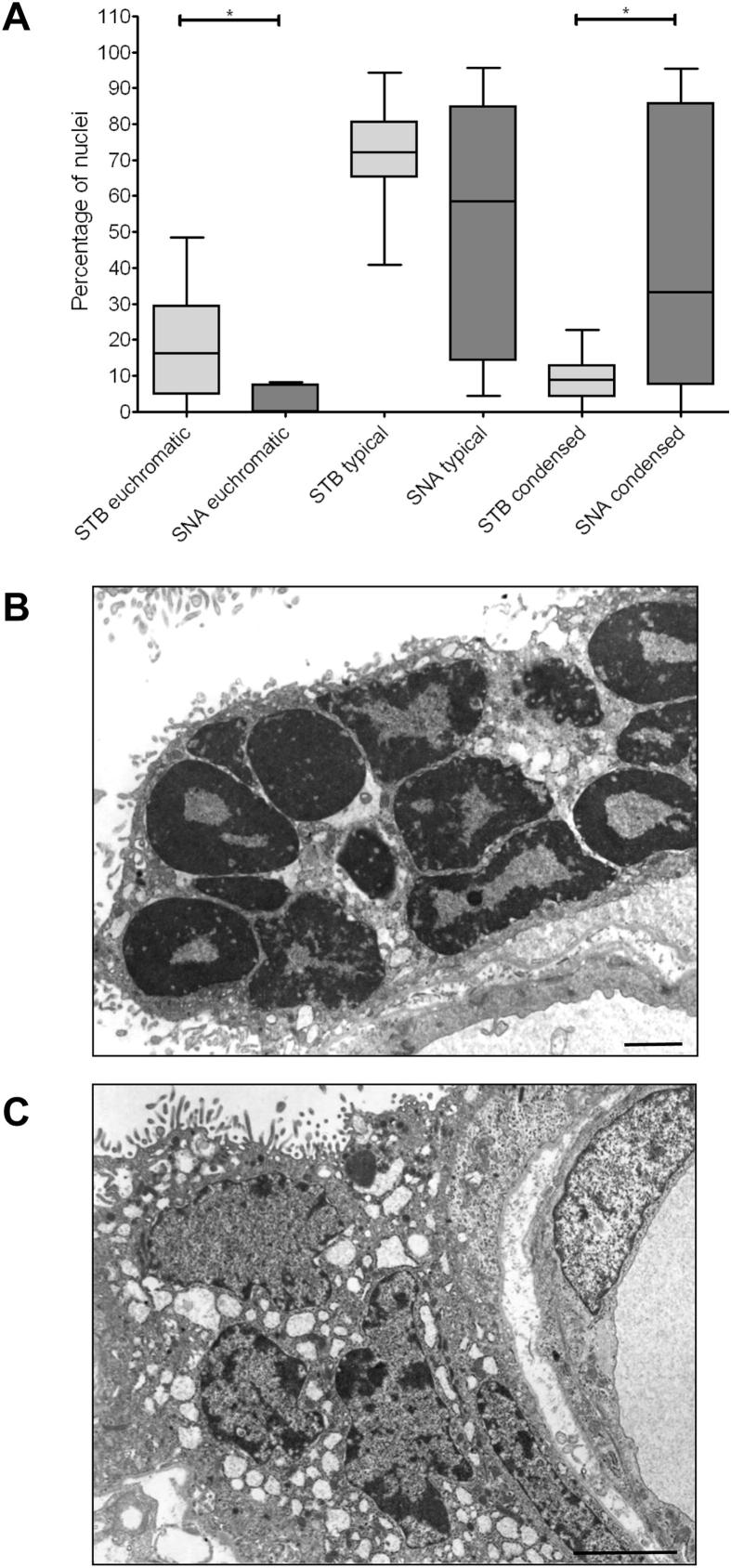
Nuclei classified for chromatin patterning showed significantly fewer euchromatic nuclei and significantly more condensed nuclei within SNAs compared to the syncytiotrophoblast generally (A). Examples of electron micrographs are given, (B) showing condensed nuclei in an SNA and (C) “euchromatic” and typical nuclei in the syncytiotrophoblast. *P ≤ 0.05 as assessed by Kruskal–Wallis followed by Dunn's multiple comparison test. STB: syncytiotrophoblast, scale bars: 20 μm.

Nuclei imaged by electron microscopy were assessed based on the amounts of heterochromatin as shown in Fig. 1. Review of electron micrograph archive images found a higher proportion of euchromatic nuclei in the syncytiotrophoblast compared to nuclei within SNAs. Conversely, there were an increased number of condensed nuclei within SNAs compared to nuclei throughout the syncytiotrophoblast (Fig. 3A). Fig. 3B shows condensed nuclei within an SNA, in contrast, Fig. 3C shows typical nuclei and a euchromatic nucleus.

**Fig. 4:**
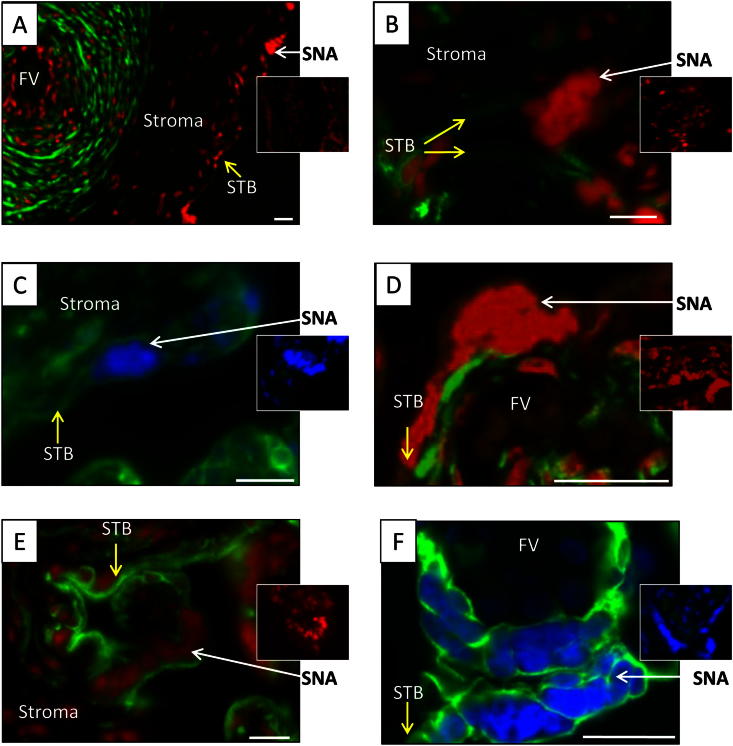
Immunofluorescence stained green for anti-α actin (A), anti-β actin (B), anti-γ actin (C), anti-β tubulin (D), anti-α tubulin (E) and anti-cytokeratin 7 (F) with respective negative controls as inserts. Nuclei were counterstained with DAPI (blue) or PI (red). White arrows: SNAs, yellow arrows: syncytiotrophoblast (STB). FV: Fetal vessels. Scale bars: 20 μm.

Cytoskeletal proteins were found throughout the syncytiotrophoblast layer and were only occasionally differently expressed around SNAs. Actin was rarely associated with SNAs; α-actin was found surrounding blood vessels (Fig. 4A), β-actin was largely expressed within villous stroma (Fig. 4B) and γ-actin (Fig. 4C) in the stroma and trophoblast layer. In contrast, α- and β-tubulin were seen throughout the syncytiotrophoblast layer but also close to SNAs just above or below prominent aggregates (Fig. 4D and E). Cytokeratin 7 was consistently found in the syncytiotrophoblast interweaving and surrounding nuclei and this was also true for SNAs (Fig. 4F).

**Fig. 5:**
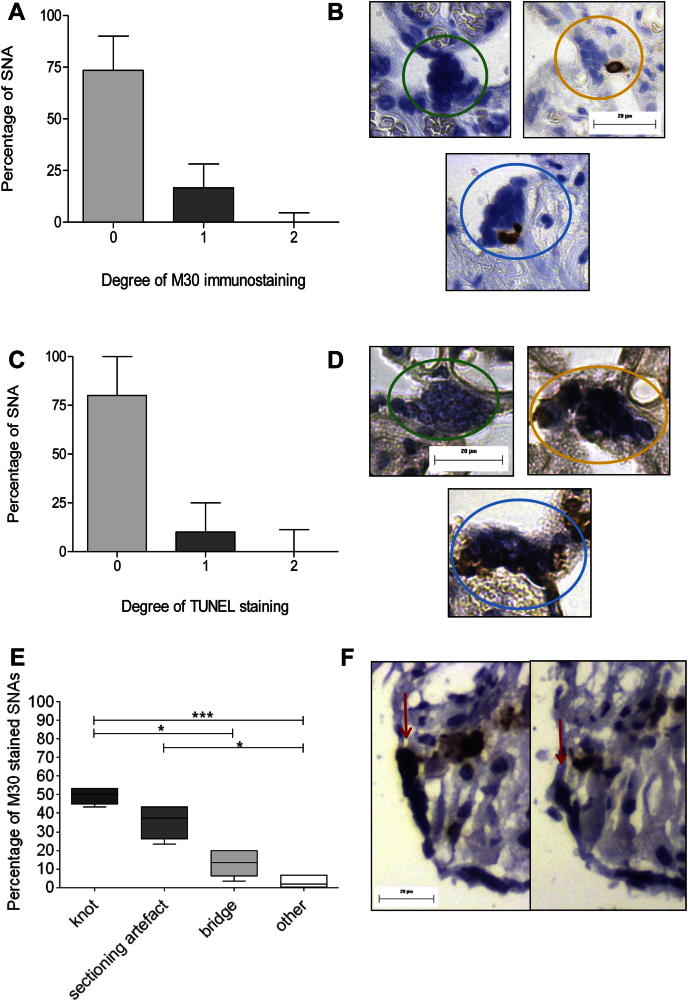
SNAs mostly failed to stain for M30-neoepitope (A) and TUNEL (C); examples of positive staining are given in B and D. Green circle: no staining, orange circle: weak staining, blue circle: strong staining. (E) Distribution of M30-positive SNAs into categories *P ≤ 0.05, ***P ≤ 0.001, as assessed by Kruskal–Wallis followed by Dunn's multiple comparison test. (F) M30-neoepitope positive SNA tracked through serial sections and revealed to be a knot (red arrow).

Most SNAs lacked staining for cytokeratin M30-neoepitope and/or TUNEL; a few had weak staining, and very few had strong M30-neoepitope or TUNEL staining (Fig. 5A and C). M30-positive SNAs traced through serial sections were more likely to be knots than bridges *P* ≤ 0.05, however knots were not significantly more likely to be M30-positive than sectioning artefacts, *P* ≥ 0.05 (Fig. 5E). Other/unclassified were significantly less likely to be M30-positive in comparison to knots and sectioning artefacts.

## 4 Discussion

This study confirms that in normal human term placenta, both syncytial knots and syncytial bridges are found. Nuclei in SNAs are morphologically different to nuclei in the remaining syncytiotrophoblast, with a higher incidence of condensed nuclei, but with little difference in cytoskeletal organisation compared to syncytiotrophoblast in general as assessed by immunofluorescence. Also, despite the appearance of nuclei in SNAs, apoptotic cell death was rare and, when present, was seen more in syncytial knots than bridges.

Sectioning artefacts comprised approximately 80% of apparent SNAs in single sections, whereas syncytial bridges and syncytial knots were similar in number and formed the majority of the remaining SNAs. These results are in agreement with Burton [19,39] and confirm the necessity to study SNAs in serial sections where possible.

When imaged by electron microscopy, SNAs were found to contain fewer euchromatic and more condensed nuclei than other areas of the syncytiotrophoblast, possibly a sign of ageing [5,6]. It is possible that SNAs could attract more heterochromatic nuclei or SNA formation could accelerate nuclear degeneration; more studies are required to discern whether either of these possibilities occurs.

Cytoskeletal proteins may have a role in SNA formation or stabilisation. Cytokeratin was heavily distributed in and around SNAs which suggests that SNAs are stable structures. The presence of tubulin indicates a potential role in SNA formation, whereas actin is less likely to be involved as it was not noticeably associated with SNAs. On the other hand several genes of proteins associated with the cytoskeleton are repressed during syncytialisation including microtubule-associated protein 4 and keratin-7, -15 and -18 [40], which could explain why some proteins are found more commonly in stroma or cytotrophoblasts rather than in the syncytiotrophoblast.

M30-neoepitope and TUNEL were found infrequently in syncytiotrophoblast or SNAs, being found in only 16.6% and 10.0% of apparent SNAs in single sections, respectively. This estimate appears greater than the levels of apoptosis described by Smith et al. [3] who found only 0.26% of nuclei in the term placenta were TUNEL-positive. However, the definition of an SNA as containing at least 10 clustered nuclei means that these values should be reduced at least 10-fold for comparison. If this adjustment is applied, the level of apoptosis in SNAs is consistent with other studies. In addition, anti-apoptotic proteins Mdm2, Bcl-2, XIAP and survivin are not reduced in the region of SNAs [41]. Combined, these data suggest that apoptosis is not a prerequisite for nuclear inclusion into SNAs.

Importantly, the level of apoptosis varied between different types of SNA which may relate to different roles. Syncytial bridges, which are infrequently apoptotic, are hypothesised to provide an internal strut system that may reduce villous injury in adverse conditions such as maternal hypertension [15,42]. Their formation may account for the previously unexplained presence of adhesion molecules in the syncytial microvillous membrane [43]. Although syncytial knots were the most likely to be apoptotic of “true” SNAs, there was no greater degree of apoptosis in them than in the syncytiotrophoblast as seen in sectioning artefacts. This provides a further indication that SNAs do not have increased levels of apoptosis compared to the remaining syncytiotrophoblast. These findings thus suggest that i) SNAs do not arise from caspase-mediated apoptosis and ii) different types of SNA have varied roles within the term placenta.

The M30-neoepitope is a relatively stable product of caspase-mediated cytokeratin 18 cleavage associated with early apoptosis. Therefore, positive staining found in SNAs could be retained from apoptosis that occurred before cytotrophoblast fusion rather than during SNA formation, or *de novo* proteolysis triggered within an SNA. It should be noted that caspase activation occurs within skeletal muscle cells to allow those cells to fuse and therefore caspases may also have a role in cytotrophoblast fusion rather than exclusively as a programmed cell death marker [44]. If caspase-mediated apoptosis is not occurring, then autophagy, another regulated process that can lead to cell death, could be contributing towards the syncytiotrophoblast nuclear clustering [45] by elimination of areas of cytosol.

Caspase-mediated apoptosis has been shown to occur primarily within cytotrophoblasts or in the syncytiotrophoblast next to fibrin deposits [31,46], so apoptotic effectors are not free to diffuse within the syncytiotrophoblast layer. This phenomenon may account for the positive apoptosis markers found in this paper in the syncytiotrophoblast. In addition, Fogarty et al. and Ellery et al. [28,47] have shown transcription in the syncytiotrophoblast layer and partially within SNAs, so nuclei in SNAs may contribute to placental function. These observations call into question the model of syncytiotrophoblast turnover [20–23] where nuclear features of apoptosis commence with cytotrophoblast fusion and continue until effete nuclei are aggregated into syncytial knots prior to being shed into the maternal circulation.

The increased number of SNAs in single sections of preeclamptic placentas has been attributed to a greater number of sectioning artefacts because of a more branched placental structure [48]. This does not account for the increased numbers of SNAs in IUGR where villous branching is reduced [49]. Further work is needed to clarify why SNAs are increased in pregnancy complications and whether the proportions of sectioning artefacts, knots and bridges differ between normal and complicated pregnancies.

SNAs may form to structurally reinforce the placenta and minimise damage from shear stresses or other mechanical sources, reduce the proportion of nuclei in highly active vasculo-syncytial membranes or result from cell turnover in the placenta without an apoptotic trigger or shedding process. Ultimately, a better understanding of the processes leading to SNA formation will give insight into their significance in pregnancy complications.
