# Supplementary material for: Syncytial nuclear aggregates in normal placenta show increased nuclear condensation, but apoptosis and cytoskeletal redistribution are uncommon
Source: Placenta. 2013 May;34(5):449–55. doi: 10.1016/j.placenta.2013.02.007 (PMC3661987; doi:10.1016/j.placenta.2013.02.007)
Supplement: Supplementary file 1 [file mmc1.ppt]

## Slide 1
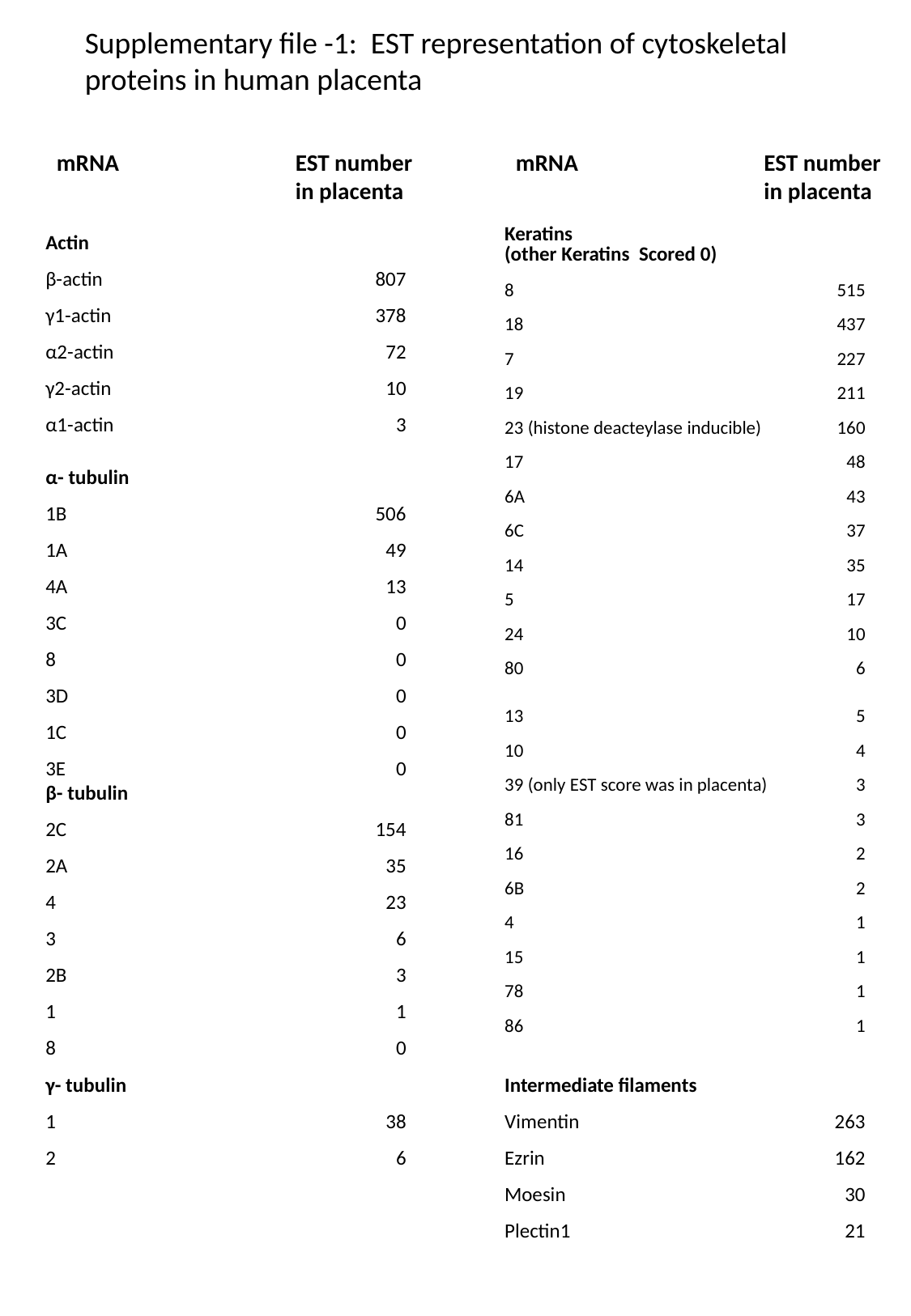

Supplementary file -1: EST representation of cytoskeletal proteins in human placenta
mRNA
EST number in placenta
mRNA
EST number in placenta
| Actin | |
| --- | --- |
| β-actin | 807 |
| γ1-actin | 378 |
| α2-actin | 72 |
| γ2-actin | 10 |
| α1-actin | 3 |
| Keratins (other Keratins Scored 0) | |
| --- | --- |
| 8 | 515 |
| 18 | 437 |
| 7 | 227 |
| 19 | 211 |
| 23 (histone deacteylase inducible) | 160 |
| 17 | 48 |
| 6A | 43 |
| 6C | 37 |
| 14 | 35 |
| 5 | 17 |
| 24 | 10 |
| 80 | 6 |
| 13 | 5 |
| 10 | 4 |
| 39 (only EST score was in placenta) | 3 |
| 81 | 3 |
| 16 | 2 |
| 6B | 2 |
| 4 | 1 |
| 15 | 1 |
| 78 | 1 |
| 86 | 1 |
| α- tubulin | |
| --- | --- |
| 1B | 506 |
| 1A | 49 |
| 4A | 13 |
| 3C | 0 |
| 8 | 0 |
| 3D | 0 |
| 1C | 0 |
| 3E | 0 |
| β- tubulin | |
| --- | --- |
| 2C | 154 |
| 2A | 35 |
| 4 | 23 |
| 3 | 6 |
| 2B | 3 |
| 1 | 1 |
| 8 | 0 |
| γ- tubulin | |
| --- | --- |
| 1 | 38 |
| 2 | 6 |
| Intermediate filaments | |
| --- | --- |
| Vimentin | 263 |
| Ezrin | 162 |
| Moesin | 30 |
| Plectin1 | 21 |
