# Supplementary material for: Syncytial nuclear aggregates in normal placenta show increased nuclear condensation, but apoptosis and cytoskeletal redistribution are uncommon
Source: Placenta. 2013 May;34(5):449–55. doi: 10.1016/j.placenta.2013.02.007 (PMC3661987; doi:10.1016/j.placenta.2013.02.007)
Supplement: Supplementary file 2 [file mmc2.pdf]

```
import kivy
kivy.require('1.0.9') # replace with your current kivy version !

from kivy.app import App
from kivy.graphics.transformation import Matrix
from kivy.uix.slider import Slider
from kivy.uix.button import Button
from kivy.uix.label import Label
from kivy.uix.widget import Widget
from kivy.uix.scatter import Scatter
from kivy.uix.image import Image
from kivy.uix.popup import Popup
from kivy.core.window import Window
from kivy.uix.floatlayout import FloatLayout
from kivy.uix.gridlayout import GridLayout
from kivy.uix.boxlayout import BoxLayout
from kivy.uix.anchorlayout import AnchorLayout
from kivy.graphics import Color, Ellipse, Line
from kivy.uix.filechooser import FileChooserIconView

from cPickle import load, dump
from kivy.config import Config

from os import listdir
from datetime import datetime

width = 1024
height = 768

classesToColours = {
   'knot' : (1,1,0,0.5),
   'bridge' : (0,0,1,0.5),
   'other' : (1,0,0,0.5),
   'sectioning_artifact' : (0,1,1,0.5),
   'cant_tell' : (0.5,0.7,0.8,0.5),
   'unclassified' : (0.1,0.1,0.1,0.5)
}


class MyPaintWidget(Widget):
    def drawSelf(self):
        with self.canvas:
            d = 30.
            Ellipse(pos=(self.x, self.y), size=(d, d))
    def setClass(self, classification):
        self.colour = classesToColours[classification]
    def setInactive(self):
        self.canvas.clear()
        with self.canvas:
            Color(self.colour[0], self.colour[1], self.colour[2],
self.colour[3])
        self.drawSelf()
    def setActive(self):
        self.canvas.clear()
        with self.canvas:
            Color(1, 0, 1, 0.5)
        self.drawSelf()

class Point():
    def __init__(self, pos):
        self.scatter = Scatter(size_hint=(0.05,0.05), do_rotation=False,
do_scale=False)
        self.widget = MyPaintWidget()
        self.widget.setClass('unclassified')
        self.widget.setActive()
        self.scatter.x = pos[0]
        self.scatter.y = pos[1]
        self.scatter.add_widget(self.widget)
```

```
        def on_move(ob, touch):
            ob.x = min(max(ob.x, -10), ob.parent.size[0] + 10)
            ob.y = min(max(ob.y, -10), ob.parent.size[1] + 10)
        self.scatter.bind(on_touch_up=on_move)



    def setOnPress(self, callback):
        self.scatter.bind(on_touch_down=callback)

    def setInactive(self):
        self.widget.setInactive()
    def setActive(self):
        self.widget.setActive()

    def setClassification(self, classification):
        self.classification = classification
        self.widget.setClass(classification)

    def setPos(self, pos):
        self.scatter.x = pos[0]
        self.scatter.y = pos[1]

    def getDisplay(self):
        return self.scatter

    def getPos(self):
        return (self.scatter.x, self.scatter.y)


class Track():
    def __init__(self, activecallback):
        self.points = {}
        self.active = False
        self.setOnActive(activecallback)

    def deletePoint(self, layer):
        if (len(self.points) > 0):
            if not (self.points.has_key(layer)):
                return False
            if self.points.has_key(layer - 1) and self.points.has_key(layer +
1):
                return False
            del self.points[layer]
            return True
        return False

    def addPoint(self, point, layer):
        if (len(self.points) > 0):
            if (self.points.has_key(layer)):
                return False
            if not(self.points.has_key(layer - 1) or self.points.has_key(layer +
1)):
                return False
        if (self.points.has_key(layer - 1)):
            point.setPos(self.points[layer - 1].getPos())
        if (self.points.has_key(layer + 1)):
            point.setPos(self.points[layer + 1].getPos())
        if (self.active):
            point.setActive()
        else:
            point.setInactive()
        point.setOnPress(self.onActiveCallback)
        self.points[layer] = point
        return True
    def getClassification(self):
        if len(self.points) == 0:
            return None
```

```
        return self.classification

    def setClassification(self, classification):
        self.classification = classification
        for point in self.points.values():
            point.setClassification(classification)

    def setOnActive(self, callback):
        def wrapped(ev,touch):
            callback(self)
        self.onActiveCallback = wrapped

    def getPointForLayer(self, layer):
        if (self.points.has_key(layer)):
            return self.points[layer]
        return None
    def setActive(self):
        self.active = True
        for point in self.points.values():
            point.setActive()
    def setInactive(self):
        self.active = False
        for point in self.points.values():
            point.setInactive()

class Layer():
    def __init__(self, img):
        self.points = []
        self.src = img
        self.layout = FloatLayout(size=(width-150, height))
        self.layout.add_widget(Image(source=img, scale=0.1))
        self.setTransform(Matrix())
    def getSource(self):
        return self.src
    def addPoint(self, point):
        self.points.append(point)
        self.layout.add_widget(point.getDisplay())
    def deletePoint(self, point):
        self.points.remove(point)
        self.layout.remove_widget(point.getDisplay())
    def getContents(self):
        return self.layout
    def setTransform(self, transform):
        self.transform = transform
    def getTransform(self):
        return self.transform




class Menu:
    def __init__(self):
        self.layout = FloatLayout(size=(200,height), pos_hint={'x':.0,
'y':0.20})
        self._buttonYPos = 0
        size = (0.1,0.1)
        def newbutton(text):
            button = Button(text=text, size_hint=size,pos_hint={'x':.0,
'y':.1*self._buttonYPos})
            self.layout.add_widget(button)
            self._buttonYPos += 1
            return button

        self.addTrackButton = newbutton('New Track')
        self.addPointButton = newbutton('New Point\nOn Track')
        self.deletePointButton = newbutton('Delete Point')
        self.setClassButton = newbutton('Set Class')
        self.setClassButton.bind(on_press=self.classificationMenu)
```

```
        self.findUnclassified = newbutton('Find\nUnclassified')
        self.showStatsButton = newbutton('Statistics')
        self.saveButton = newbutton('Save')

    def classificationMenu(self, *args):
        layout = BoxLayout(orientation='vertical')
        popup = Popup(title='Classify',
            content=layout,
            size_hint=(None, None), size=(400, 600))
        def newbutton(text, classification):
            button = Button(text=text)
            button.bind(on_press=self.setClass(classification, popup.dismiss))
            layout.add_widget(button)
        """
           'knot'
           'bridge'
           'other'
           'sectioning_artifact'
           'cant_tell'
           'unclassified'
        """
        newbutton("Can\'t tell", 'cant_tell')
        newbutton("Knot", 'knot')
        newbutton("Bridge", 'bridge')
        newbutton("Sectioning artifact", 'sectioning_artifact')
        newbutton("Other", 'other')
        popup.open()




    def onShowStats(self, callback):
        self.showStatsButton.bind(on_press=callback)

    def setClass(self, classification, onComplete=None):
        def wrapped(*args):
            self.onSetClassCallback(classification)
            if (onComplete):
                onComplete()
        return wrapped

    def onSetClass(self, callback):
        self.onSetClassCallback = callback

    def onNewPoint(self, callback):
        def wrapped(*args):
            callback()
        self.addPointButton.bind(on_press=wrapped)
    def onDeletePoint(self, callback):
        def wrapped(*args):
            callback()
        self.deletePointButton.bind(on_press=wrapped)
    def onFindUnclassified(self, callback):
        def wrapped(*args):
            callback()
        self.findUnclassified.bind(on_press=wrapped)

    def onSave(self, callback):
        def wrapped(*args):
            callback()
        self.saveButton.bind(on_press=wrapped)

    def onNewTrack(self, callback):
        def wrapped(*args):
            callback()
        self.addTrackButton.bind(on_press=wrapped)
```

```
    def getContents(self):
        return self.layout


class MyApp(App):

    def build(self):
        Window.bind(on_key_down=self.on_key_down)
        self.loadData()
        self.activeTrack = None
        self.currentLayer = 0
        self.appstructure = FloatLayout()
        width, height = Window.size
        self.menu = Menu()
        self.menu.onNewTrack(self.newTrack)
        self.menu.onNewPoint(self.newPoint)
        self.menu.onDeletePoint(self.deletePoint)
        self.menu.onSetClass(self.setClass)
        self.menu.onFindUnclassified(self.jumpToUnclassified)
        self.menu.onShowStats(self.showStats)
        self.menu.onSave(self.save)
        self.core = Scatter(auto_bring_to_front=False)
        self.core.add_widget(self.getCurrentLayer().getContents())
        self.appstructure.add_widget(self.core)
        self.appstructure.add_widget(self.menu.getContents())
        self.zoomSlider = Slider(orientation='vertical', min=1, max=10,
size_hint=(0.05,1),pos_hint={'x':0.95})
        self.zoomSlider.bind(on_touch_move=self.on_touch_move)
        self.zoomSlider.bind(on_touch_down=self.on_touch_down)
        self.zoomSlider.bind(on_touch_up=self.on_touch_up)
        self.appstructure.add_widget(self.zoomSlider)
        self.imagelabel = Label(text=self.getCurrentLayer().getSource(),
size_hint=(1,0.05), pos_hint={'y':0})
        self.appstructure.add_widget(self.imagelabel)
        self.zooming = False
        return self.appstructure


    def loadImages(self):
        def isImage(fname):
            for ext in [".png", ".jpg", ".tiff", ".jpeg", ".bmp"]:
                if fname.lower().endswith(ext):
                    return True
            return False

        self.layers = []
        for img in sorted(listdir("images")):
            if isImage(img):
                self.layers.append(Layer("images/"+img))

    def loadData(self):
        self.loadImages()
        self.tracks = []
        try:
            trackreps = load(open("saveFile.data"))
        except:
            return
        for trackrep in trackreps:
            track = Track(self.setActive)
            for pointrep in trackrep['points']:
                point = Point(pointrep[1])
                track.addPoint(point, pointrep[0])
                self.layers[pointrep[0]].addPoint(point)
                point.setPos(pointrep[1])
            track.setClassification(trackrep['classification'])
            track.setInactive()
            self.tracks.append(track)
```

```
    def save(self):

self.saveTo("backup-"+datetime.today().strftime("%Y-%m-%d-%H-%M-%S")+".data")
        self.saveTo("saveFile.data")

    def saveTo(self, fileName):
        #save to file ...
        def trackRepresentation(track):
            trackrep = {'classification':track.getClassification()}
            trackrep['points'] = []
            for layer in range(len(self.layers)):
                point = track.getPointForLayer(layer)
                if point:
                    trackrep['points'].append((layer, point.getPos()))
            return trackrep

        savetracks = []
        for track in self.tracks:
            savetracks.append(trackRepresentation(track))
        print savetracks
        dump(savetracks, open(fileName, 'w+'))

    def on_touch_down(self, slider, ev):
        if (slider.collide_point(ev.pos[0], ev.pos[1])):
            self.zooming = True

    def on_touch_move(self, slider, ev):
        if (self.zooming):
            zoom = self.zoomSlider.value
            self.core.scale = zoom

    def on_touch_up(self, slider, ev):
        self.zooming = False

    def showStats(self, *args):
        classCounts = {}
        for classification in classesToColours:
            classCounts[classification] = 0
        for track in self.tracks:
            if track.getClassification():
                classCounts[track.getClassification()] += 1

        text = ""
        for classification in classCounts:
            text +=  "    %s : %s
\n"%(classification,classCounts[classification])
        popup = Popup(title='Statistics',
            content=Label(text=text),
            size_hint=(None, None), size=(400, 400))
        popup.open()

    def setClass(self, classification):
        if (self.activeTrack):
            self.activeTrack.setClassification(classification)

    def setActive(self, track):
        if (self.activeTrack):
            self.activeTrack.setInactive()
        self.activeTrack = track
        track.setActive()
    def newPoint(self):
        if (self.activeTrack == None):
            return
        point = Point((width/2,height/2))
        if (self.activeTrack.addPoint(point, self.currentLayer)):
            self.getCurrentLayer().addPoint(point)
    def deletePoint(self):
        if (self.activeTrack == None):
```

```
            return
        point = self.activeTrack.getPointForLayer(self.currentLayer)
        if (point == None):
            return
        if not(self.activeTrack.deletePoint(self.currentLayer)):
            return
        self.getCurrentLayer().deletePoint(point)
        if (self.activeTrack.getClassification() == None):
            self.tracks.remove(self.activeTrack)
            self.activeTrack = None


    def newTrack(self):
        track = Track(self.setActive)
        point = Point((width/2, height/2))
        track.addPoint(point, self.currentLayer)
        track.setActive()
        track.setClassification('unclassified')
        self.tracks.append(track)
        self.setActive(track)
        self.getCurrentLayer().addPoint(point)
    def getCurrentLayer(self):
        return self.layers[self.currentLayer]

    def jumpToUnclassified(self):
        for track in self.tracks:
            if track.getClassification() == 'unclassified':
                for i, layer in enumerate(self.layers):
                    if track.getPointForLayer(i):
                        self.setActive(track)
                        self.swapLayer(self.getCurrentLayer(), layer)
                        self.currentLayer = i
                        return
        popup = Popup(title='',
            content=Label(text="No unclassified tracks found!"),
            size_hint=(None, None), size=(400, 400))
        popup.open()



    def moveUpLayer(self):
        if (self.currentLayer < (len(self.layers) - 1)):
            original = self.getCurrentLayer()
            self.currentLayer += 1
            new = self.getCurrentLayer()
            self.swapLayer(original, new)
    def moveDownLayer(self):
        if (self.currentLayer > 0):
            original = self.getCurrentLayer()
            self.currentLayer -= 1
            new = self.getCurrentLayer()
            self.swapLayer(original, new)

    def swapLayer(self, old, new):
        if (old == new):
            return
        self.imagelabel.text = new.getSource()
        self.core.add_widget(new.getContents())
        self.core.remove_widget(old.getContents())
        # current transform is layer * user
        layer = new.getTransform()
        transform = self.core.transform
        user = transform.multiply(layer.inverse())
        # put back to start
        self.core.apply_transform(self.core.transform_inv)
        # apply layer transformation
        self.core.apply_transform(old.getTransform())
        # reapply user transformation
```

```
        self.core.apply_transform(user)
    def on_key_down(self, instance, code, *args):
        if (code == 275):
            self.moveUpLayer()
        if (code == 276):
            self.moveDownLayer()

if __name__ in ('__android__', '__main__'):
    print Config.get('graphics','width')
    MyApp().run()
```
